# Health Promotion as a Motivational Factor in Alpine Cycling

**DOI:** 10.3390/ijerph18052321

**Published:** 2021-02-26

**Authors:** Marco Haid, Elisabeth Nöhammer, Julia N. Albrecht, Alexander Plaikner, Harald Stummer, Peter Heimerl

**Affiliations:** 1 Division for Management in Health and Sport Tourism, UMIT Tirol—Private University for Health Sciences, Medical Informatics and Technology, 6060 Hall in Tirol, Austria; peter.heimerl@umit.at; 2 Institute for Management and Economics in Healthcare, UMIT Tirol—Private University for Health Sciences, Medical Informatics and Technology, 6060 Hall in Tirol, Austria; elisabeth.noehammer@umit.at (E.N.); harald.stummer@umit.at (H.S.); 3 Department of Tourism, University of Otago, Dunedin 9054, New Zealand; julia.albrecht@otago.ac.nz; 4 Department of Strategic Management, Marketing and Tourism, University of Innsbruck, 6020 Innsbruck, Austria; alexander.plaikner@uibk.ac.at

**Keywords:** motives, alpine region, self-determination theory

## Abstract

The present study examines motives for cycling in the alpine region and focuses on the relative importance of health promotion with respect to other motives. Furthermore, the influences of person-specific characteristics on the rank of the motives are examined, and possibilities for advertising bike tourism based on these motives and characteristics are derived. By applying a quantitative approach, a total of 175 cyclists were surveyed using questionnaires on person-specific characteristics, motives, and their relevance for alpine cycling. Data analysis revealed that health promotion is the most important motive for alpine cycling after fun and action as well as nature experience. Further health-related motives such as stress reduction are also perceived as important. The social component, on the other hand, was given the least priority. The results also showed that person-specific characteristics influence the relative importance of motives. For example, elderly persons and people with children perceive the motive of health promotion as the most important. The study shows that the health-promoting effect of alpine cycling is noticed and may be further encouraged. This study demonstrates that alpine cyclists are a heterogeneous group and that health benefits are perceived by various sub-groups therein. Therefore, any marketing for alpine cycling needs to reflect the diversity of cyclists, and approaches need to be adapted according to the respective target group.

## 1. Introduction

Increasing work pressure and performance requirements lead to increased stress, which has a significant role in the development and progression of fatigue as well as physical and mental illness [1,2]. The resulting direct costs (e.g., therapies and medical treatments) and indirect costs (e.g., absenteeism and reduced work performance) have a significant economic impact, which is why stress reduction and health promotion are becoming increasingly important [3]. Nature and time spent outdoors can play a decisive role in promoting health and well-being [4,5,6,7,8,9], even simply through exposure to them [10]. Physical activities also provide health benefits and result in improved wellbeing [7,11,12], especially when performed outdoors [7,13,14]. Thus, outdoor activities are particularly beneficial to health [7,11,15,16].

Cycling is an outdoor activity that is becoming increasingly diffused in the alpine region, an area with a great potential for health benefits due to its unique and rich nature [17,18,19]. This sport contributes significantly to health and wellbeing, for both active and inactive individuals [7,20,21,22,23,24]. Its current popularity is especially due to the various possibilities of cycling in the Alps and the advanced e-bike technology [19,25] that facilitates access to the sometimes difficult terrain.

This study covers all forms of cycling in the alpine region, its nature, and environment, which are included in alpine cycling. Due to the various possibilities advertised, it considers different target groups (e.g., sportive adventurers, families, more senior citizens) as well as all forms of cycling such as mountain biking, e-biking, and even road cycling on alpine streets [17,26,27,28].

The motivation to exercise or be physically active has been the subject of numerous investigations [29,30,31,32,33]. With regard to cycling motivations, previous studies found experience of nature, escape from everyday life, physical exercise, recreation, and fun followed by maintaining good health as the dominant motives [18,34,35]. Other relevant factors are the availability of a good cycling infrastructure, the aim of reducing one’s carbon footprint, self-presentation, or social factors [36,37].

Although studies have already been conducted on specific types of cycling, such as mountain biking, or in non-alpine regions, there are no studies known to the authors regarding cycling motives in the alpine region in particular (except for a master thesis [38]). Therefore, open questions are whether there is a health motive in alpine cycling and if so, how it relates to other motivational factors, and which other motives and personal characteristics are relevant for alpine cycling. The purpose of this study was to investigate the importance of health promotion in alpine cycling compared to other motives and to examine the influence of person-specific factors. It also aimed at determining which motives and characteristics the advertisement of cycle tourism can be based on. Drawing on these findings, the health-promoting effects of alpine cycling could be better strengthened and integrated into health promotion recommendations.

This study applied the self-determination theory (SDT) as a theoretical basis. SDT is frequently used to understand motivation in the fields of sports and exercise [33,39,40]. It is one of the most important theoretical approaches towards the study of motivation [41,42] and is used, among others, to investigate the motivation to exercise of different groups of people [43,44,45,46,47], to investigate the characteristics of exercise goals [39,48], and, more generally, to study the motives for sports and exercise [40,49,50,51,52]. According to the SDT, experiencing competence or achievement (competence), personal/social connection (relatedness), and autonomy are drivers for high-quality motivation and are considered as “basic psychological needs” [50,53,54]. Competence relates to feeling able to actively interact with the environment and achieve a desired goal [55]; relatedness concerns the desire for social interaction, while autonomy refers to the ability to autonomously and freely decide what is best for oneself [55]. Building on the basic needs, the SDT locates motivation on a continuum between intrinsic motivation and amotivation [33,42,56]. The former is defined as doing an activity because of its inherent satisfaction or enjoyment, leading to the highest degree of motivation, and the latter as the lack of any kind of motivation to engage in an activity [42,50]. Between the two extremes lies extrinsic motivation, subdivided into four types of behavioral regulation along a continuum from non-autonomous to fully autonomous forms: (1) external, a type of regulation with low autonomy, which indicates an activity that is performed only because of external rewards or to satisfy the needs of others; (2) introjected, indicating an activity performed to avoid experiencing guilt or shame relating to internal pressures; (3) identified, related to an activity performed because of the benefits associated with it; (4) integrated, the most autonomous form, associated with an activity performed because the reasons underlying it have been integrated into the individual itself [42,50,56,57,58]. The major difference between highly autonomous extrinsic motivation types and intrinsic motivation is that the activity itself is not perceived as inherently pleasurable in the extrinsic variants. In addition, extrinsic motivation is defined as doing an activity for instrumental reasons or for achieving an outcome that can be separated from the activity itself [50]. Based on the SDT, we assumed that in alpine cycling, the intrinsic motives fun and action derived from the activity (i.e., pleasure and enjoyment of the activity) have the highest values in relation to other motives. Hence, we derived the following hypotheses:

**Hypothesis** **1** **(H1).** *Fun and Action in cycling are the most important motives in alpine cycling.*

Further, experience of nature as an intrinsic motive is supposed to play a major role in opting for alpine cycling a sporting activity. The unique nature of the alpine region and its experience are an inseparable, essential, and inherent part of alpine cycling, as this activity takes place in the alpine environment and cannot, for example, occur indoors or on the home trainer. However, it could be achieved by means of other activities (e.g., hiking), which is why we expect it to be second to fun and action.

**Hypothesis** **2** **(H2).** *Nature experience is the second most important intrinsic motivation in alpine cycling.*

Intensive and fulfilling training is indicated as a possible extrinsic motivation because it is an instrumental reason or result of alpine cycling that can be reached without this specific activity [50]. However, it is postulated as a highly important motivating factor as cycling in an alpine region can be expected to be more strenuous than in other surroundings. Since the degree of autonomy it involves is high and it strengthens and requires competence, we expect it to be the most important extrinsic motivation. However, intrinsic motivation is postulated to have a more important effect than extrinsic aspects, so we estimate it to rank after these. Thus, we propose:

**Hypothesis** **3** **(H3).** *Intensive and fulfilling training is the most important extrinsic motive in alpine cycling but ranks after intrinsic aspects.*

Other extrinsic motives are health promotion as well as related motives (coping with stress, switching off from everyday life), as they, too, can be separated from the activity itself and make it the means to reach a goal. A high degree of autonomy can also be assumed for these motives, as well as the requirement and strengthening of competence. However, we assume increased external determination (e.g., to reduce health risks) and thus less autonomy than for intensive and fulfilling training. We, therefore, derived the following hypothesis:

**Hypothesis** **4** **(H4).** *Health promotion is the second most important extrinsic motive in alpine cycling, also ranking after intrinsic aspects.*

Since alpine cycling is a sport that can be performed alone, we do not expect social reasons to play a major role but tested them as a control (see Methods section below).

Being a popular sport, cycling attracts people with different characteristics. E-bike riders, for example, are particularly interested in social contact, environmental awareness, and the promotion of health [59]. In many aspects of cycling, gender-specific differences suggest that women are affected differently by certain factors (e.g., bicycle facilities, safety) [60]. Over the life course, the access to and reason for using the bike change [61]. These findings motivated further investigation of personal characteristics. Hence, we derived the following hypothesis:

**Hypothesis** **5** **(H5).** *Person-specific characteristics influence the relative importance of motives for alpine cycling.*

The great potential of cycling tourism is highlighted in many tourism and strategy papers [17]. Especially, new bike technologies (e-bike) offer great opportunities for tourism [18,19]. The majority of bicycle tourists have a high level of education, a high income, and thus a high economic potential and high relevance for tourism [34]. So far, however, few studies have been carried out to examine the tourism promotion strategies for cycling in general and in the alpine region in particular. Providing insight into the motives and characteristics of cyclists on whom the promotion of alpine cycling can be based was thus also a purpose of this study.

## 2. Materials and Methods

### 2.1. Research Design and Administration

For this quantitative, correlational, and cross-sectional study [62], data were collected in May and June 2020 using a questionnaire. The questionnaire was subjected to two rounds of pilot testing before being administered to research participants. The research project was advertised to several local and regional biking groups through a variety of social media channels. Interested individuals were encouraged to participate in an online survey. The questionnaire was in German and divided into three main areas: (1) an introduction explaining the reason for the study and the questionnaire, (2) a survey of motives for cycling in the Alps, and (3) a survey of personal characteristics of the cyclists. The motives were measured with a 7-point Likert-scale from 1 “no agreement” to 7 “absolute agreement”. The cyclists’ personal characteristics were collected with selection questions and open-text entries.

The motives for alpine cycling were measured as single items in the questionnaire, a common strategy in studies on cycling [18,21,34,35,59,63]. Despite concerns about content validity, reliability, and sensitivity, this is considered adequate and even advantageous over multiple-item measurements [64,65,66,67,68,69,70], especially if the items used are sufficiently narrow and unambiguous, and are collected in the form or type of single ease questions [69,71,72,73]. With regard to the SDT, as intrinsic motivation, “Action and fun while cycling” (AF) was queried in addition to “Nature experience” (NE). For extrinsic motives, based on the research hypotheses and inspired by previous studies, “Health promotion” (HP), “Stress reduction” (SR), “Escaping from everyday life” (EEL), “Intensive and fulfilling training” (IFT), and “Activity with other people” (AOP) were included in the questionnaire. On the basis of the SDT, the impact of the extrinsic motivations mentioned is influenced by the degree they fulfill basic psychological needs. HP, for instance, is driven more by autonomy and competence, while relatedness is more important for AOP.

As personal characteristics, the following items were queried, inspired by previous studies [18,19,34,60,61]: gender, age, origin, marital status, number of children, e-bike or classical bike, alternative sports.

### 2.2. Sample

One hundred eighty-six questionnaires were returned, 11 were excluded due to missing data on motives [74,75]. Therefore, a total of 175 data sets was collected and analyzed. Of these sets, 64 (36.6%) were provided by females, and 111 (63.4%) by males; the average age was 32.9 years, and on average, the participants had 1.13 children. In addition, 54 (30.9%) of the participants were single, 73 (41.7%) were in a partnership, 45 (25.7%) were married, and 3 participants did not provide any information in this regard. Twenty-eight (16%) of the respondents stated they rode an e-bike, 159 (84%) reported the use of other non-motorized bicycle types. Of the 175 participants, 87 (49.1%) were from Austria, 61 (34.86%) from Germany, and 25 (14.3%) from South Tyrol and other northern Italian regions. Only one person was from Switzerland, and one from another European country. In total, 25 (14.3%) participants had an income below €500, 33 (18.9%) reported an income between €500 and €1499, 46 (26.3%) reported an income between €1500 and €2499, while 18 (10.3%) have an income above €2500 and below €3500 (all values are net, i.e., after taxes and charges). Fifty-threee (30.3%) participants provided no information on their income.

### 2.3. Data Analysis

The data analysis was performed using frequency analysis, the location measures mean and median, standard deviation, and interquartile range. Furthermore, due to data scaling, correlation analysis was performed using Spearman’s rank-order correlation, and the non-parametric Mann–Whitney U and Kruskal–Wallis H test were used to investigate significant differences [76]. Group comparisons were done regarding bicycle types (e-bike vs. non-motorized bike), gender (female vs. male), and marital status (single vs. married/in partnership).

## 3. Results

### 3.1. Motives for Alpine Cycling

The most important motive for alpine cycling was “action and fun while cycling”, followed by “nature experience” and “health promotion”. The least important iwa “activity with other people”. Table 1 lists all motives examined.

Table 2 shows the correlations of the motives in a correlation analysis. “Health promotion” had a significantly positive correlation with the thematically close motive “stress reduction”. The most remarkable significant positive correlation of “health promotion” was found with “intensive and fulfilling training”. Also, the “experience of nature” showed a significant positive correlation with “health promotion”. “Stress reduction” and “escape from everyday life” had a particularly high positive correlation (rs = 0.79).

### 3.2. Cyclist Characteristics and Motives

#### 3.2.1. Bicycle Type

**Figure 1:**
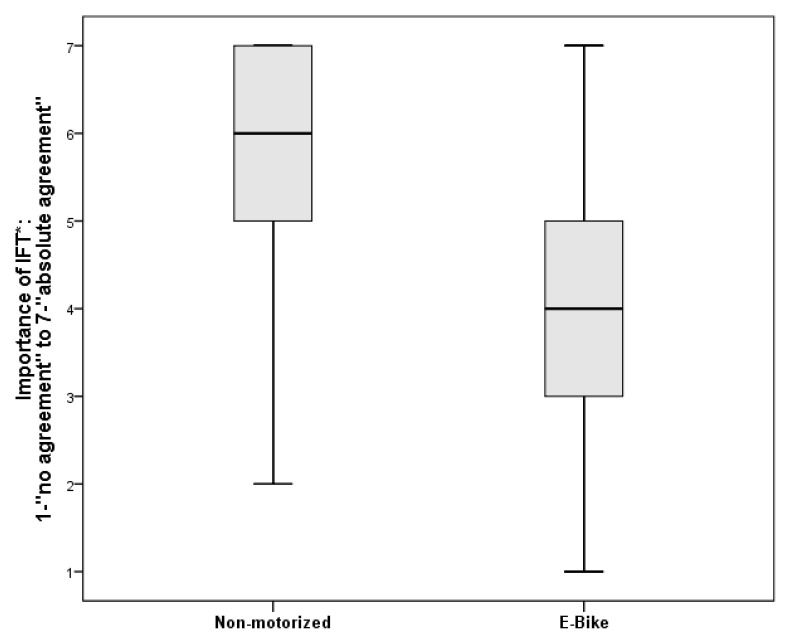
Comparison with boxplots: IFT (Intensive and fulfilling training)-Non-motorized vs. E-bike.

The motive “intensive and fulfilling training” was significantly less important for people who rode e-bikes (mean rank = 49.73) than for participants who rode non-motorized bikes (mean rank = 95.29), U = 986.5, *p* = 0.00 (Figure 1). Apart from that, there were no significant differences between bike types with regard to motives.

#### 3.2.2. Gender, Age, Marital Status, and Children

**Figure 2:**
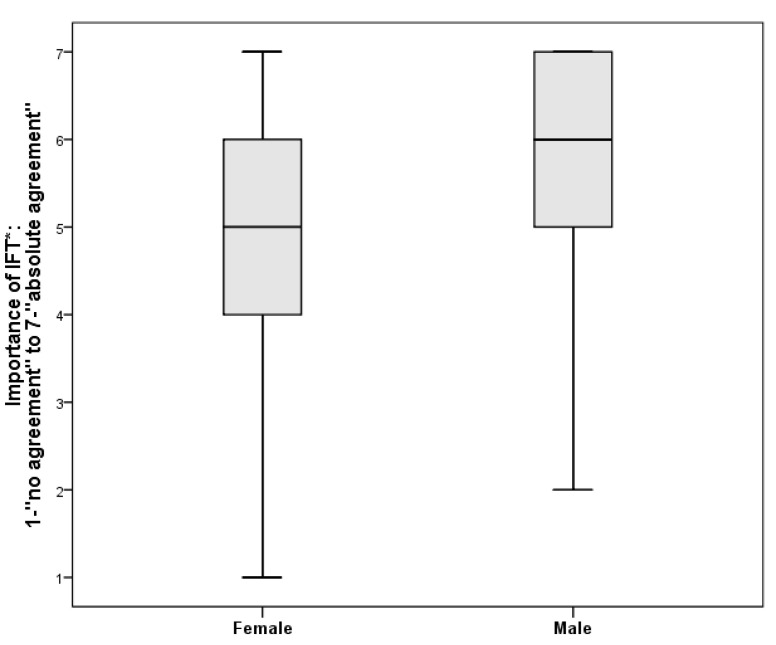
Comparison with boxplots: IFT (Intensive and fulfilling training)-Male vs. Female.

Female participants rated the motive “Intensive and fulfilling training” as significantly less important (mean rank = 72.89) than men (mean rank = 96.71), U = 4519.00, *p* = 0.002 (Figure 2). No other gender-specific differences in motives were found.

A higher age correlated significantly positively with the motives “health promotion” (rs = 0.170) and “experience of nature” (rs = 0.066).

The motive “Action and fun while cycling” appeared more important for singles (mean rank = 93.31) than for people in partnerships (mean rank = 91.22) or for married people (mean rank = 70.67), H = 7,051, *p* = 0,029. The family situation also influenced other motives: the number of children correlated significantly negatively with the “Action and fun while cycling” (rs = −0.205), but showed a significant positive correlation with the motive “Health promotion” (rs = 0.254).

#### 3.2.3. Alternative Sports

The majority of people surveyed practiced at least one alternative sport in both summer and winter. In summer, hiking was the most frequently mentioned (119 mentions), before running (97 mentions), mountain tours (75), and climbing (56 mentions). In winter, skiing (108 mentions), ski touring (82 mentions), and snowboarding (37 mentions) were the favored alternative sports before tobogganing (32 mentions) and snowshoeing (19 mentions). The participants did not use the available free-text field in the questionnaire for entering other sports.

## 4. Discussion

In the course of the study, motives for alpine cycling were examined.

Individuals may have multiple forms of motivation for a particular activity, exhibiting both intrinsic motivation (e.g., they find pleasure and enjoyment in it) and extrinsic motivation (e.g., it is necessary for the achievement of a desired state) [77,78]. Due to this, a sporting activity, for instance, can be either only intrinsically motivated (e.g., it is done because of its pleasure and enjoyment), only extrinsically motivated (for this, the degree of autonomy is essential)—for instance, to reduce health risks—or motivated by both intrinsic and extrinsic aspects.

All motives studied were rated relatively high for alpine cycling (values above 4 on the seven-level Likert scale). The highest rating was given to “Action and fun while cycling while cycling”. Hence, the collected data support hypothesis H1. According to the SDT, pleasure can be classified as an inherent satisfaction associated with the activity itself and hence as an intrinsic motivational factor [50,79]. “Action and fun while cycling” can, therefore, be considered as a particularly sustainable motivational factor. “Experience of nature” was the second-highest motive, hence the data also support hypothesis H2. Experiencing nature is an inherent aspect of alpine cycling, though it could, of course, also be achieved through other activities, which is why it is classified as an extrinsic motivation in other contexts [50,80,81]. In an Alpine context, supporting these intrinsic motives is rather easy. “Intensive and fulfilling training” can be categorized as an extrinsic motive, as it is an outcome and can be separated from alpine cycling itself [50]. Contrary to our assumptions, it was not the most important extrinsic motive and ranked markedly behind “Health promotion” and related motives. Therefore, hypothesis H3 was rejected.

“Health promotion”, a particularly positive effect of cycling [20,21] and extrinsic motive was rated the third highest. Thus, H4 was rejected: contrary to our expectations, health promotion was not the second but the prime extrinsic motivation. This result highlights the heterogeneity of alpine cyclists and challenges the widespread opinion that alpine cycling is only attracting (young) people looking for a strenuous, adrenaline-rich mountain bike challenge. The data indicate that alpine cycling is attractive due to many more reasons and thus for a wider audience.

“Health promotion” is strongly connected to “Escaping from everyday life” (the fourth highest) and to “Stress reduction” (the fifth highest). This is in line with a study by Wolf and Seebauer [59], which shows that health is an important motive for leisure cycling. Regarding duration, reducing health risks (e.g., high cholesterol levels) as a motive might only persist until this aim is reached, whilst a desire to promote health, for example, to stay fit, would be more associated with long-term motivation [50,53]. As alpine cycling allows for a high degree of autonomy, which according to the SDT is a driver for sustainable long-term motivation overall and for individual motives [50,79], this activity is an ideal candidate for health promotion. Accordingly, it can be assumed that health promotion is pursued autonomously [50].

“Health promotion” is also related to “Intensive and fulfilling training”, in addition to “Stress reduction”. In turn, “Stress reduction” is related to “Experience of nature” and, very strongly, to “Escape from everyday life”. The potential of nature to create new experiences, switch off from everyday life, and reduce stress has already been demonstrated in previous work [8].

Although “Activities with other people” was rated the least important, the mean value of the ratings was still above the middle of the seven-point Likert scale. According to the SDT, personal or social connection is an important driver for sustainable motivation [50,79] and thus also an important cornerstone for alpine cycling. However, the individual experience of nature, sports, and action might be more important in motivating people to engage in alpine cycling.

Respondents who used an e-bike rated the motive “Intensive and fulfilling training” significantly less important. According to Wolf and Seebauer [59], the social context, ecological convictions, and health are important motivating factors for e-bike riders. It can be assumed that for e-bike riders, “Intensive and fulfilling training” is no focal aim since it cannot be reached via the chosen means, suggesting that the comfort of the activity is more relevant.

There were gender-specific differences in the reasons and motives for riding a bicycle [60]. In the alpine context, a significant difference could only be found in one of the motives studied: women considered the motive “Intensive and fulfilling training” less important than men. Reasons and motives for cycling also change over the life-course [61]. Consistent with this, the results in this study also point to age-related differences: with increasing age, the motives “Health promotion” and “Experience of nature” seem to become more important.

A further influencing factor of the value of the alpine cycling’s motives is the family situation: first, the results showed that for singles, “Action and fun while cycling” were significantly more important than for individuals in partnerships or for married persons. Second, there was a negative correlation with “Action and fun while cycling”. Third, the value of the motive “Health promotion” was positively related to the number of children. A possible explanation for this would be that having children might lead to increased health awareness and desire for health promotion.

The data collected show that personal characteristics influence the relative importance of motives for alpine cycling. Therefore, hypothesis H5 was supported.

The results showed that, in addition to cycling, other sports are practiced both in summer and in winter. The most frequent mentions regarded sports close to nature and mountain-oriented. From this, it can be deduced that the affinity for nature and alpine sports is also valid outside of cycling and that the preferences and motives collected could also be crucial for other activities and their promotion.

This study has some limitations. Due to the cross-sectional design, no interpretations of causal mechanisms can be made based on the data. Since a quantitative approach was employed, deeper content-related insights into the effects and interactions of the various motives were only possible to a limited extent. Moreover, the sample was not randomly chosen to represent a certain population. By using contacts to cycling groups and also by employing an online questionnaire, a bias may have been introduced. While this strategy allows a higher likelihood of reaching people engaged in alpine cycling, former or potential future users might not have been covered, as well as more elderly cyclists without internet access. This was partly alleviated by the fact that alpine cycling is expected to rather attract the younger population.

## 5. Conclusions

The present study examined the importance of the motive “Health promotion” with regard to other motives and personal characteristics, indicating that alpine cycling is attractive to various target groups for various reasons. This study offers insights into the motives and characteristics on which the promotion of bike tourism in the alpine region can be based. The study’s contribution is threefold: first, it contributes to the literature dealing with health promotion in the context of sports. Second, it expands the knowledge of motivational factors in sports with the application of the SDT. Third, for practitioners, the study provides ideas on how to promote alpine cycling activities based on motivational factors.

Due to the health benefits of sports and alpine cycling, in particular, having more people engaging in it presents individual and public health benefits. Moreover, an active lifestyle should be maintained over the life course. Thus, promotional messages for alpine cycling should highlight the health benefits, like the maintenance of vitality and physical aesthetics for more senior users. For younger cyclists, health promotion must be adapted accordingly in service communication. Messages could convey that health is the basis for intensive training and key to reaching specific spots like peaks. The approach to reach young cyclists should also be probably more community-based and use various (social) media. More traditional channels such as catalog advertising or circulars may still be the most advantageous for older cyclists. However, social media should not be underestimated regarding older cyclists, but the right platforms have to be chosen.

Due to the heterogeneity of the cyclists and their motives, the promotion of alpine cycling must be strongly aligned with the respective target groups. In general, action and fun while cycling and the experience of nature are important for every group and should be focused on, also for promoting other sports activities in the region. To attract men, training intensity and the effects of alpine cycling should be highlighted, whereas for families and the elderly, health promotion is more relevant. For the latter, it is important to know that a variety of cycles, including e-bikes, is offered or possible to use. Regarding younger people, mentioning the effects of stress reduction due to alpine cycling seems promising. The social component and the possibility of performing an activity with other people showed the least importance for all groups and should thus not play a prominent role in promoting the activity.

Future research should further examine both from a medical and from a tourist point of view how alpine cycling possibilities can and must be designed in order to achieve the best possible effect on public health and the sustainable development of tourism destinations. Based on the presented data, the high affinity with nature and the pleasure derived from the activity could be used to alleviate seasonal differences. For example, summer cycling could be intensively promoted in ski resorts. The well-attended winter season could thereby act as an advertising platform for the summer, promote summer tourism, and contribute to a sustainable destination development.

## Figures and Tables

**Table 1:** Descriptive statistics.

Measures		AF	NE	HP	SR	EAL	IFT	AOP
Mean		6.0514	5.9143	5.6609	5.5771	5.5829	5.2914	4.0629
Median		6.0000	6.0000	6.0000	6.0000	6.0000	5.0000	4.0000
Std. Deviation		1.06279	1.18349	1.31903	1.49079	1.47887	1.46244	1.77840
Minimum		2.00	1.00	1.00	1.00	1.00	1.00	1.00
Maximum		7.00	7.00	7.00	7.00	7.00	7.00	7.00
Percentiles	25	5.0000	5.0000	5.0000	5.0000	5.0000	4.0000	3.0000
	50	6.0000	6.0000	6.0000	6.0000	6.0000	5.0000	4.0000
	75	7.0000	7.0000	7.0000	7.0000	7.0000	6.0000	5.0000

Legend: AF = Action and fun while cycling, NE = Nature experience, HP = Health promotion, SR = Stress reduction, EAL = Escaping from everyday life, IFT = Intensive and fulfilling training, AOP = Activity with other people.

**Table 2:** Correlations, Spearman Rho: Motives.

		NE	HP	SR	EAL	IFT	AOP
**AF**	Correlation Coefficient	0.081	0.125	0.390 **	0.309 **	0.338 **	0.123
	Sig. (2-tailed)	0.285	0.100	0.000	0.000	0.000	0.105
	*n*	175	174	175	175	175	175
**NE**	Correlation Coefficient		0.259 **	0.139	0.149 *	0.039	0.128
	Sig. (2-tailed)		0.001	0.066	0.049	0.611	0.092
	*n*		174	175	175	175	175
**HP**	Correlation Coefficient			0.232 **	0.221 **	0.452 **	−0.129
	Sig. (2-tailed)			0.002	0.003	0.000	0.091
	*n*			174	174	174	174
**SR**	Correlation Coefficient				0.790 **	0.292 **	0.022
	Sig. (2-tailed)				0.000	0.000	0.770
	*n*				175	175	175
**EAL**	Correlation Coefficient					0.265 **	−0.041
	Sig. (2-tailed)					0.000	0.594
	*n*					175	175
**IFT**	Correlation Coefficient						−0.092
	Sig. (2-tailed)						0.225
	*n*						175

Legend: AF = Action and fun while cycling, NE = Nature experience, HP = Health promotion, SR = Stress reduction, EAL = Escaping from everyday life, IFT = Intensive and fulfilling training, AOP = Activity with other people. * Correlation was considered significant at the 0.05 level (2-tailed). ** Correlation was considered significant at the 0.01 level (2-tailed).

## Data Availability

The data presented in this study are openly available in FigShare at https://doi.org/10.6084/m9.figshare.14123849, [82].
